# Infection dynamics in a traveller with persistent shedding of Zika virus RNA in semen for six months after returning from Haiti to Italy, January 2016

**DOI:** 10.2807/1560-7917.ES.2016.21.32.30316

**Published:** 2016-08-11

**Authors:** Luisa Barzon, Monia Pacenti, Elisa Franchin, Enrico Lavezzo, Marta Trevisan, Dino Sgarabotto, Giorgio Palù

**Affiliations:** 1 Department of Molecular Medicine, University of Padova, Padova, Italy; 2 Microbiology and Virology Unit, Padova University Hospital, Padova, Italy; 3 Transplant Infectious Disease Unit, Padova University Hospital, Padova, Italy

**Keywords:** emerging or re-emerging diseases, imported viral diseases, laboratory surveillance, molecular methods, vector-borne infections, sexually transmitted infections

## Abstract

We describe the dynamics of Zika virus (ZIKV) infection in a man in his early 40s who developed fever and rash after returning from Haiti to Italy, in January 2016. Follow-up laboratory testing demonstrated detectable ZIKV RNA in plasma up to day 9 after symptom onset and in urine and saliva up to days 15 and 47, respectively. Notably, persistent shedding of ZIKV RNA was demonstrated in semen, still detectable at 181 days after onset.

A patient, who developed fever and rash after returning from Haiti to Italy, was diagnosed with Zika virus (ZIKV) infection in January 2016. Longitudinal follow-up laboratory testing was performed to characterise ZIKV RNA and antibody dynamics during acute infection. A relevant finding in this case was the persistent shedding of ZIKV RNA in semen for six months after symptom onset.

## Case report

In January 2016, a man in his early 40s returning to Italy from a two-week stay in Haiti developed fever (38.5 °C) and pruritic maculopapular rash on his trunk and arms that fully resolved after three days. The patient, who reported mosquito bites in Haiti, had an unremarkable past personal medical history. Laboratory analyses, performed at day 3 after symptom onset, showed blood cell count and liver function tests within the normal range. Testing for dengue, chikungunya and ZIKV infection, according to previously described methods [1], demonstrated the presence of ZIKV RNA in plasma and urine at 175 copies/mL and 25,600 copies/mL, respectively, and ZIKV-specific IgM but not IgG antibodies. Dengue virus (DENV) IgG antibodies were also detected by ELISA, but they represented cross-reacting antibodies induced by previous vaccination against yellow fever virus, as confirmed by virus neutralisation assays; DENV IgM, DENV NS1 antigen and chikungunya virus IgM and IgG were negative. Sequencing of the full ZIKV genome was obtained directly from a urine sample collected at diagnosis (GenBank KX269878), which demonstrated over 99.6% nucleotide sequence identity with ZIKV strains circulating in Haiti (GenBank KU509998 and KX051563).

## Follow-up evaluation

Based on these findings, a diagnosis of ZIKV infection was made. The patient was informed about the risk of sexual transmission of ZIKV and was advised to adopt safer sex practices. Further laboratory testing was performed at five days post onset of symptoms, which demonstrated the presence of ZIKV RNA also in saliva (58,700 copies/mL) and semen (175 copies/mL), while stool samples and a conjunctiva swab were negative. The patient did not report haematospermia or prostatitis. The patient was invited to participate in a follow-up evaluation of ZIKV RNA kinetics in various bodily fluids and of ZIKV-specific antibodies in serum. During follow-up, saliva and urine samples were collected daily, while blood and semen samples were collected at least weekly. Follow-up visits for clinical evaluation and counselling were performed weekly. Follow-up is still ongoing at the time of this report, with the latest evaluation performed on day 181 after symptom onset.

During follow-up, laboratory testing (Figure) demonstrated that viral RNA was detectable in his plasma at low titre (ca 100 copies/mL) up to day 9 after symptom onset. Viral load in urine was higher than in blood (ca 25,000 copies/mL), but rapidly decreased to undetectable levels at two weeks after symptom onset. Shedding of ZIKV RNA in saliva persisted up to day 47, at a median load of 400 copies/mL (range: 80–3,300), after peak values of 20,000–50,000 copies/mL during the first week after symptom onset. It is noteworthy that ZIKV RNA shedding in semen was sustained and persistent, and still detectable at day 181 after symptom onset. In particular, after a peak of ca 50,000 copies/mL at day 14, viral RNA load in semen was stable in consecutive specimens, ranging from 1,000 to 10,000 copies/mL (Figure, panel A). Separation by centrifugation of cellular and plasma fractions showed that viral RNA was associated with the cellular component of semen, while undetectable in seminal plasma. Positive ZIKV real-time RT-PCR results were confirmed by repeat testing and by analysis with alternative methods, i.e. a LightMix Modular Zika Virus kit (Roche Diagnostics, Basel, Switzerland), broad-range pan-flavivirus RT-PCR followed by Sanger sequencing [2] and Sanger sequencing of the viral genome.

**Figure:**
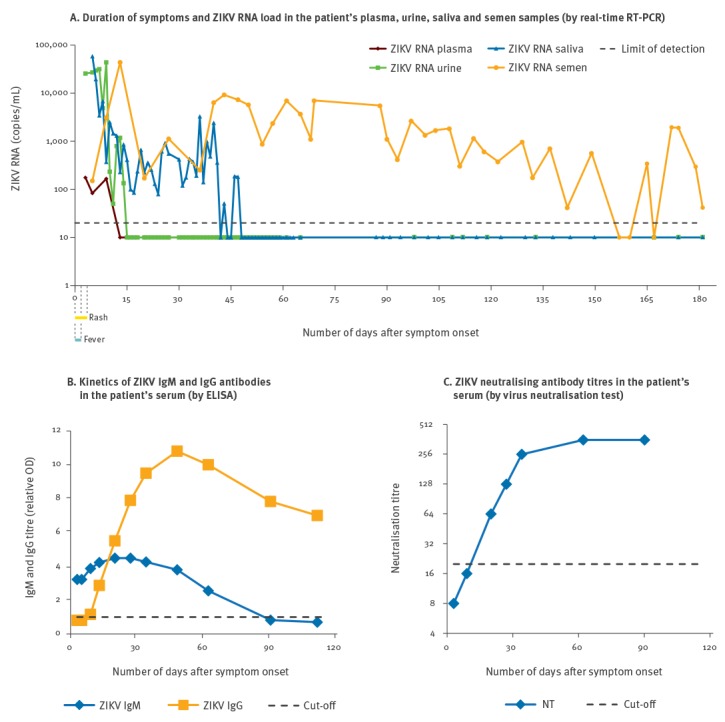
Clinical and laboratory findings in a patient with Zika virus infection returning from Haiti to Italy, January 2016

OD: optical density; ZIKV: Zika virus; NT: neutralisation titre.

Virus isolation in cell culture was attempted with ZIKV RNA-positive serum, urine, saliva and semen specimens collected within the first two weeks after symptom onset, but no infectious ZIKV was recovered.

Finally, ZIKV IgG antibodies appeared at 13 days after symptom onset, while IgM antibodies were already present at the time of the first evaluation at day 3 and become negative at day 90 (Figure, panel B). Neutralising antibodies were detected at day 20 and reached a peak titre of 1:358 at day 62 (Figure, panel C).

## Background

Zika virus is an emerging mosquito-borne flavivirus that has spread in the Americas. By early February 2016, 500,000 to 1,500,000 cases of ZIKV disease were estimated to have occurred in Brazil since the beginning of the outbreak [3]. Association of the infection with Guillain–Barré syndrome and fetal microcephaly led the World Health Organization to declare the 2015–16 outbreaks of ZIKV infection in the Americas a public health emergency of international concern [4]. Besides mosquito-borne transmission, several cases of sexual transmission of the virus have been documented, related to viral shedding in semen [5-7]. Cases with prolonged shedding of ZIKV in semen have been reported, up to 62 days [8], 76 days [7] and 93 days [9] after symptom onset. Infectious virus has been recovered in semen up to 24 days [5] and cases of sexual transmission occurring weeks after the index case have been described [10]. Detection of ZIKV RNA in vaginal fluids and cervical mucus during acute infection has been reported [11], indicating a potential risk for female-to-male sexual transmission.

## Discussion

A remarkable aspect of this case was the long duration of viral nucleic acid shedding in semen (still detectable in semen at 181 days after symptom onset). Moreover, testing of serial samples allowed us to characterise the pattern of ZIKV shedding in semen and other bodily fluids during the course of infection. In addition to semen, viral RNA was detectable for a long period also in saliva, as previously described in another patient [1], notwithstanding the rapid induction of ZIKV-specific IgM and IgG antibodies and high-titre neutralising antibodies. The mechanisms of ZIKV persistence in the human host, the cellular reservoirs involved, as well as the mechanisms of viral clearance are still unknown and should be investigated.

Since ZIKV infection may be transmitted through sexual intercourse [5-7], data from this case suggest a prolonged potential for sexual transmission. However, the presence of ZIKV RNA in semen does not imply the presence of infective virus – it could just represent a trace of past infection. 

The results of this study may have potential implications for preconception counselling recommendations. According to the current recommendation of the United States Centers for Disease Control and Prevention, men who have had a diagnosis of ZIKV disease and do not reside in an area with active ZIKV transmission should wait for at least six months after symptom onset before attempting conception [12]. This interval was recommended based on information regarding persistence of ZIKV RNA in semen thus far available and allowed for three times the longest period that ZIKV RNA had been detected in semen after symptom onset (62 days) [12]. Similar recommendations have been released by WHO, which advise male travellers returning from areas of known ZIKV transmission to adopt safer sex practices and wait at least eight weeks (six months if symptomatic) before trying to conceive [13]. At the light of this new evidence on long-term ZIKV RNA persistence in semen, an extension of this interval might be considered or ZIKV RNA testing in semen after the eight-week or six-month period might be advised.

The pattern of ZIKV shedding in semen is largely unknown [14]. In the case reported here, longitudinal sampling showed continuous shedding of ZIKV RNA at a stable and relatively high load. This finding would support ZIKV RNA testing is semen samples to detect infection, as proposed also by WHO guidance [13]. However, if a first result is negative, testing of at least an additional semen sample should be recommended before excluding infection, because of the risk of false-negative results, as shown in the case described here (Figure, panel A).

The risk of sexual transmission of ZIKV seems to be associated with excretion of ZIKV at high viral load during the early phase of infection [5,8,15,16], but cases of late sexual transmission [10] as well as transmission between asymptomatic individuals [17] have been also reported. In the case described here, ZIKV RNA load was low in semen samples collected three months after symptom onset. Transmission with such a low level of ZIKV RNA in semen has not been established, but cannot be ruled out. Thus, due to the limited available information, as a precautionary measure when issuing recommendations, the risk of transmission through sexual intercourse or gamete donation in the presence of low-level ZIKV nucleic acids in semen samples should not be overlooked, balancing the principles of precaution and proportionality. Further studies are also warranted to establish the prevalence and duration of ZIKV shedding in semen, the risk of virus transmission through semen, and the cells targeted by ZIKV infection in the genital tract.
